# Creating the Cave: Conducting Circadian Science in Early Childhood

**DOI:** 10.3390/clockssleep5010009

**Published:** 2023-02-20

**Authors:** Lauren E. Hartstein, Sachi D. Wong, Leen Abbas, Sophia Choubai, Jonah N. Wilson, Trace Jablin, Monique K. LeBourgeois

**Affiliations:** Department of Integrative Physiology, University of Colorado Boulder, Boulder, CO 80309, USA

**Keywords:** melatonin, circadian rhythms, children, preschool, research methods

## Abstract

In humans, physiological outputs of the body’s internal clock (i.e., saliva, serum, and temperature) can be collected to quantify the timing of the circadian system. In-lab assessment of salivary melatonin in a dimly lit environment is a common approach for adolescents and adults; however, the reliable measurement of melatonin onset in toddlers and preschoolers requires a modification of laboratory methods. For > 15 years, we have successfully collected data from ~250 in-home dim light melatonin onset (DLMO) assessments of children aged 2–5 years. Although in-home studies of circadian physiology may introduce a host of challenges and may increase the risk of incomplete data (e.g., accidental light exposure), in-home studies afford more comfort (e.g., less arousal in children) and flexibility for families. Here, we provide effective tools and strategies to assess children’s DLMO, a reliable marker of circadian timing, through a rigorous in-home protocol. We first describe our basic approach, including the study protocol, collection of actigraphy data, and strategies for training child participants to complete procedures. Next, we detail how to convert the home into a “cave”, or dim-light environment, and present guidelines for timing the salivary data collection. Lastly, we provide helpful tips to increase participants’ compliance based upon behavioral and developmental science tenets.

## 1. Introduction

To assess circadian timing in humans, researchers examine outputs of the body’s internal clock (i.e., circadian markers), such as the sleep-promoting hormone melatonin [1]. In humans, melatonin levels begin to rise in the evening as the body prepares for sleep, peak in the middle of the night, then gradually decrease throughout the second half of the night and into the morning. Melatonin can be measured directly through saliva or blood plasma or as a metabolite (6-sulphatoxymelatonin) in urine. Light is the primary time cue (i.e., zeitgeber) to the internal biological clock. Light acutely suppresses evening melatonin levels and shifts circadian timing, thus necessitating that assessments are performed under dim light conditions [2,3]. Unlike other circadian markers (e.g., cortisol, core body temperature), the circadian rhythm of melatonin is not masked by sleep or meals, and it remains stable across repeat assessments [4]. Without the need to restrict food intake and sleep, evening melatonin onset is an ideal phase marker of circadian timing in young children.

A reliable measure of circadian timing is the salivary dim light melatonin onset (DLMO) [5], which is the time at which evening melatonin levels cross a pre-determined threshold to indicate the start of the biological night [6,7,8]. In children and adults, a later DLMO is associated with later bedtimes, midsleep times, and wake times [6,9,10]. To assess DLMO, participants provide regular biological samples, often at 30 or 60 min intervals throughout the evening, under dim light conditions. This is typically performed in a laboratory setting so that the subject’s environment can be tightly controlled. However, spending extended time in a laboratory providing samples can be stressful for young children and may not be feasible for parents juggling multiple children and other time commitments. In-home assessments offer a more accessible solution for families while creating a comfortable “real-life” environment for children that is conducive to ecologically valid data. Although in-home data collection can be difficult and poses a risk of accidental exposure to light intensities outside of the study’s parameters, these risks can be mitigated through careful planning, open communication with families, and preliminary training with child participants. The goal of this paper is to provide researchers with the tools to conduct rigorous, developmentally focused in-home circadian assessments under well-controlled conditions [6,11,12].

## 2. Basic Study Protocol

**Figure 1:**
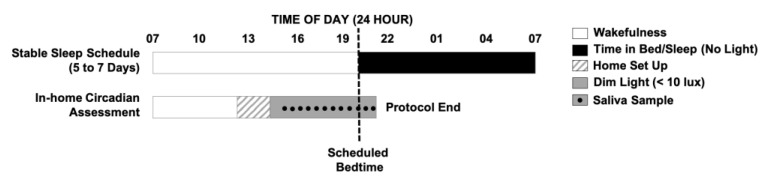
Protocol to assess DLMO. For 5 to 7 days, children follow their habitual parent-selected sleep schedule. On the day of the circadian assessment, children enter a dim-light environment 5 h and 30 min before bedtime (< 10 lux). They provide saliva samples throughout the evening at 30 min intervals until 1 h past their habitual bedtime (12 samples). The times depicted are meant as an example of a parent-selected sleep schedule and associated protocol times for an individual child participant.

To assess a baseline DLMO, participants and their families complete a 6- to 8-day protocol (Figure 1). All study procedures take place in participants’ homes where they are comfortable and can follow their usual routines. All study procedures are approved by an institutional review board before data collection and written informed consent is obtained from parents. Although inclusion/exclusion criteria for participants are unique to each research question examined with this protocol, examples of our criteria for conducting assessments with healthy children can be found in our previously published work [6,11]. The season of assessment is important to consider when conducting circadian research in order to account for differences in photoperiod. However, this protocol has been used successfully to collect data across the school year [6,11], as well as exclusively during the summer months [12,13].

### 2.1. Habitual Sleep Schedule

**Figure 2:**
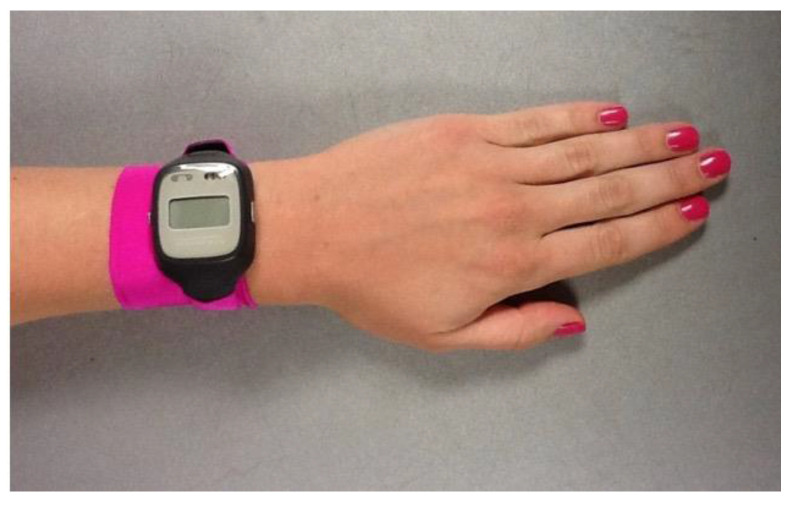
An actigraph watch secured with a Lycra band.

Throughout the study, children are required to sleep in their own bed alone each night and refrain from ingesting caffeine (e.g., chocolate, caffeinated teas and sodas, etc.) or any medication affecting sleep or circadian rhythms. For the first 5 to 7 days of the protocol, children follow their habitual sleep schedule to provide an accurate representation of their typical sleep patterns. Parents complete a daily diary to report children’s bedtimes and wake times, night awakenings, and daytime activities that may impact their sleep. Additionally, researchers make daily contact with families via telephone or text to obtain bedtimes and wake times and confirm compliance with study rules. These daily check-ins provide an opportunity for researchers to answer any questions and build rapport with families. Children also wear a wrist actigraph throughout the study [6,11], which provides measurements of objective sleep timing and light exposure. To make the actigraphs “kid-friendly”, children are provided with watch bands made of soft, elastic Lycra that make the actigraph comfortable to wear and secure on the wrist (Figure 2). 

### 2.2. In-Home DLMO Assessment

On the day of the circadian assessment, researchers transform the participant’s home into a dim-light environment (< 10 lux), playfully dubbed a “cave”, which is maintained until the study ends. Researchers remain in the home during the child’s waking hours to uphold adherence to the study protocol and maintenance of the dim-light environment, with at least one parent on the premises at all times. The researchers, known to children as the “sleep fairies”, bring games, books, and toys to engage the child participant or any interested siblings. The child enters the dim-light environment 5 h and 30 min before their average bedtime and remains in dim light through the completion of the protocol. Starting 4 h and 30 min before the child’s habitual bedtime, saliva collection begins, with samples collected at 30 min intervals until 1 h past bedtime (12 samples total). The choice of the start and end times allows us to capture low melatonin levels during the day, as well as individual differences in children’s increasing melatonin levels and DLMO, and ensures that we do not miss their DLMO, should it occur after habitual bedtime. The timing of each saliva sample is anchored to the child’s average bedtime from the previous 5 to 7 days. If actigraphy reveals a long sleep onset latency (> 30 min), saliva collection is extended for an additional 1 h to avoid a “missed” evening DLMO. This basic DLMO protocol can be modified and extended to capture morning dim light melatonin offset or to conduct experimental research, such as on the effects of evening light on circadian timing or melatonin suppression [13,14].

## 3. Saliva Collection

Children refrain from eating or drinking anything for 15 min before each sample. We communicate in advance with parents to avoid selecting foods that will get stuck in the teeth or gums (e.g., cheddar crackers and popcorn); however, if the child eats between samples, they then swish water through their teeth and chew gently on a dental cotton roll on both sides of their mouth to remove any food debris. Melatonin concentrations in both plasma and saliva are altered by changes in posture [15]; thus, subjects must maintain a consistent posture just before and during sample collection [8]. To control for postural influences, the child performs a “sit still” time, where they maintain an upright seated position for 5 min before and during each sample collection.

**Figure 3:**
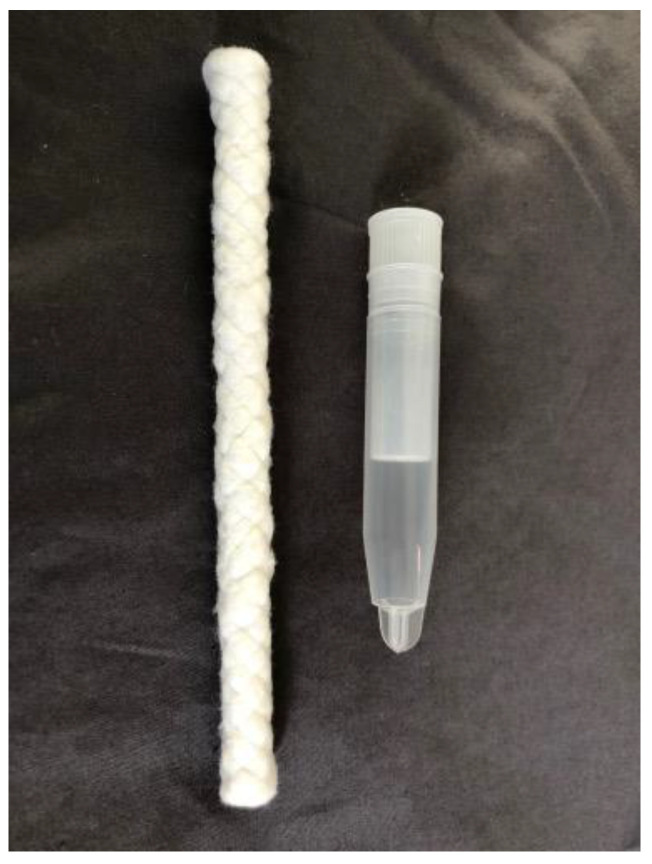
The braided dental cotton roll and tube used to collect saliva samples.

To collect saliva, researchers place one end of a braided dental cotton roll (Henry Schein Inc., Denver, PA, USA) in the child’s mouth and instruct them to chew for 1–3 min, or until the cotton roll is saturated (Figure 3). The researchers wear gloves throughout the sampling procedure and maintain a hold on one end of the cotton roll when it is in the child’s mouth to prevent a choking hazard. The dry excess of the cotton roll is then cut off with scissors, which are cleaned with alcohol wipes between each sample. The saliva-saturated cotton is inserted into a salivette tube (Sarstedt, Nümbrecht, Germany). Each tube is identified on the side with a label including the ID number, date, and sample number. Additionally, we identify the sample number on the cap of the tube. Researchers confirm aloud that the correct sample is inserted into each tube. The sample is then immediately centrifuged for ~5 min. The cotton roll and inner tube are removed and discarded, and the outer tube, now containing the saliva, is immediately stored in the family’s freezer upright in a tube rack or box. The recommended volume for each saliva sample is 2 ml to obtain an adequate quantity for repeat assays. Researchers also measure light intensity during each saliva sample at the child’s eye level by holding a research photometer ~5 cm from the child’s face and directing it in their approximate angle of gaze. For each sample, the start time, end time, light intensity, and any additional notes are recorded on a data tabulation form. After the data collection is complete, the saliva samples are transported to the laboratory in a cooler with ice packs and then transferred immediately to a −20 °C freezer.

### Analysis

**Figure 4:**
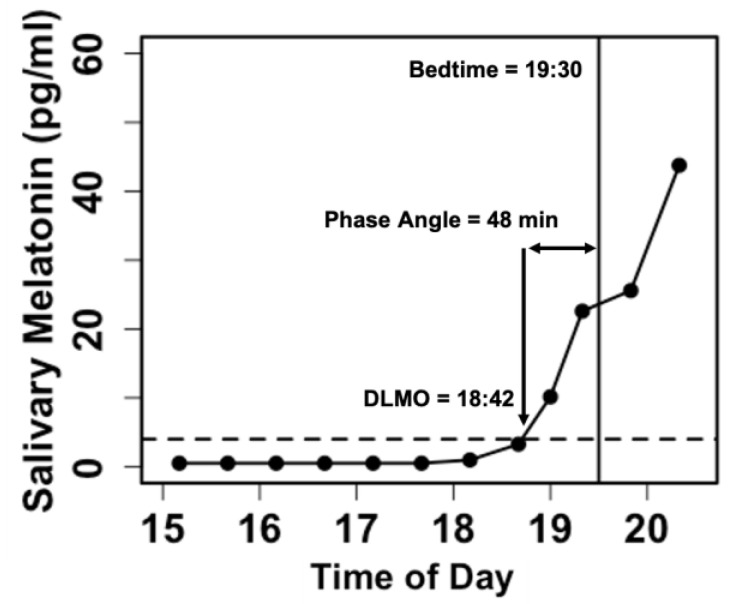
Example of a dim light melatonin curve for one individual child participant. Each black dot represents a saliva sample collected. The dotted line denotes the DLMO threshold of 4 pg/ml. In this example, the child’s scheduled bedtime (lights-out time) was 19:30. DLMO was calculated as 18:42. The phase angle difference between lights-out time and DLMO was 48 min.

Samples are assayed via radioimmunoassay (Bühlmann Laboratories AG, Schöenbuch, Switzerland) to determine the concentration of melatonin. In research on children and adolescents, DLMO is typically calculated as the linearly interpolated clock time at which salivary melatonin levels cross a threshold of 4 pg/ml [7,16] (Figure 4). DLMO determined in this manner is a reliable measure in young children and is stable across repeated assessments [6]. With our actigraphy and salivary melatonin data, we also compute the phase angle difference between lights-out time and DLMO, which may be used to assess the degree of internal circadian alignment [17].

## 4. Setting up the Dim-Light Environment (Creating the Cave)

During the initial consent visit, researchers conduct an environmental assessment of the home in order to individualize setup procedures. Researchers note the number of rooms to be set up, the approximate sizes of windows to be covered, and whether any specialized materials (e.g., a tall ladder) will be required. For a medium-sized home, 2 h are allocated to complete the dim-light setup. Upon arriving at a family’s home, researchers first download and check actigraphy to determine the habitual bedtime. The researchers then review what to expect during the setup and throughout the evening with the parent. To begin the setup, all windows and any other openings to outside light (e.g., skylights, sliding glass doors, pet doors, etc.) are covered. This is accomplished using black plastic sheeting, or “tarps”, and 3M brand painter’s tape. Certain paint types, drywall textures, and humidity may impact tape adhesion. As part of our initial environmental assessments, we test how well the tape adheres to various surfaces, such as walls and window frames, so that we can plan accordingly. Although only lightweight tarps are purchased, large tarps can still be heavy and must be checked regularly throughout the in-home protocol and reinforced when necessary. A fallen tarp poses a huge risk of unintended light exposure and, thus, a failed assessment. Signs are placed on all home entryways with reminders to call, text, or knock before entering and to ensure the child is in another room before entering or exiting the home.

Researchers then strategize where to provide light to make the space comfortable for the family while ensuring a well-controlled, dim-light environment. If a home has dimmable overhead lights, they are adjusted to the required light levels. For light fixtures that do not dim, lightbulbs may be replaced with lower-wattage bulbs or a customized-size tarp canopy may be affixed to the ceiling and draped underneath the fixture to direct the light upward. Recessed LED ceiling lights are covered with multiple layers of painter’s tape to reach the desired intensity. When using tape and tarps near lights, researchers check that the lights are not too hot and do not pose a fire risk if covered. It is of paramount importance that every switch in a home be flipped to identify what it controls. Every light or dimmer switch is then firmly taped over to avoid accidental light exposure. Similarly, unused lamps are unplugged, or their lightbulbs are removed. To supplement areas where home lights cannot be used, small dimmable lamps or LED light bars are employed. These sources are best placed on a high surface and dimmed to the necessary intensity. As light sources that are already present in the home (e.g., dimmable ceiling lights) are frequently employed in the cave, the exact type and spectrum of light that each child is exposed to during the assessment differs. The protocol only requires that the illuminance of light remains < 10 lux at the child’s angle of gaze throughout, so we do not recommend any particular type or brand of light for this protocol. During the setup process, researchers log anything that they move or unplug on a data sheet so that the home is returned to its original state at the end of the study. Our goal is to leave a house exactly how we entered it.

Although a benefit of conducting research in the home is maintaining the child’s comfort in their natural environment, every home presents unique challenges to controlling exposure to light. However, the risk of accidental bright light exposure can be greatly reduced with proper preparation, problem solving, and vigilance. Many modern appliances (e.g., refrigerator, freezer, water dispenser, oven, microwave, and clothes dryer) have automatic lights. If the machine’s light cannot be covered or disabled, the parent is instructed to communicate with researchers when they need to use the appliance. Researchers then ensure that the child is in a different room before any light-emitting source is used. Additionally, it is crucial to look for possible hidden sources of light (e.g., children’s toys, shoes, toothbrushes, flashlights, night lights, sunrise alarm clocks, smart devices, etc.). Researchers scour the home and consult with the parents and child to identify anything that may have a light. If an object is deemed too bright, it is stashed out of the child’s reach for the duration of the study. Researchers complete a final walkthrough of the home to verify that all light sources are < 10 lux from the child’s angle of gaze. Informing the parents that the child cannot be lifted vertically (e.g., while playing) during the study and that electronic devices (e.g., phone) cannot be used in the child’s vicinity also reduces the risk of light exposure. Clear communication between the researchers and the family is paramount to ensure a successful DLMO assessment.

## 5. Ensuring Compliance

In our assessments, we pay careful attention to and appreciate individual differences in what children like or do not like and how we can motivate them. Each child’s temperament and interests are different; therefore, flexibility is key for a successful assessment. For example, for some children, a sibling serves as a supportive figure and role model for behavior throughout the protocol. For others, a sibling is a distraction or source of jealousy over rewards and attention from researchers. The techniques described below provide a framework that researchers can adapt to meet the demands of each individual assessment.

### 5.1. Training

To increase the likelihood of success with sample collection, three training visits are scheduled before the in-home assessment to allow the child to become comfortable with the research team and introduce the saliva collection procedure (referred to as “chewing”). Throughout the training, children are encouraged to ask questions and tactilely engage with the saliva collection materials and procedures (e.g., touching the saliva tube and cotton stick, placing their hands on the centrifuge, called “Spinny”, to feel the vibrations). These training visits are crucial to identifying the best timing and techniques to support each child in completing a successful sample. Some children provide sufficient saliva with ease, while others may struggle to produce the required 2 ml. When introducing the procedure, researchers serve as a model by first performing a chew themselves for the child to observe. To encourage the child to chew, researchers employ strategies such as asking them to chew like their favorite animal, compete against a parent/sibling to determine who can produce the most saliva, or think about their favorite food, which also stimulates saliva production. Children are instructed to gently tap on the cotton with their teeth and not to chew too hard, which can lead to saliva being expelled into the mouth and not absorbed by the cotton. For a child with a reserved or anxious disposition, parent presence and participation can be essential to success. Child-friendly terms (e.g., sleep fairies, cave days, and chewing) are also utilized throughout the assessment to help make the researchers and protocol feel less clinical and more playful and approachable for children.

### 5.2. Rewards and Motivation

An important technique used throughout our data collection to boost compliance is positive reinforcement. We use an individualized approach, dependent on each child’s interests, to reward appropriate behavior in order to increase compliance with study procedures. Small, tangible rewards are utilized for every subject, including a sticker chart and a personalized toy, thereby employing both immediate and delayed gratification as motivation. Prior to data collection, parents are asked about their child’s likes and dislikes so that researchers can provide rewards that the child will be excited to receive. Children earn stickers throughout the evening for each saliva sample. When they have collected 12 stickers (at the conclusion of the assessment), they are allowed to select between two toys that align with their interests. With parental permission, food is also used as a reward, most often in the form of a small treat (e.g., a few jellybeans or fruit snacks) after each sample. Food rewards have the added benefit of increasing saliva production during samples when the child is encouraged to think about the treat they will soon receive.

Praise from researchers or family members can also serve as positive reinforcement for behavior. The nature of the protocol is demanding, and framing it as a “job” and praising the child for their hard work creates an encouraging environment and a feeling of internal pride. This is especially important because children may become tired towards the end of the protocol. Researchers also approach each activity with enthusiasm, as children are often influenced by the tone set by the team. Some children are motivated by attention from and quality time with the researchers. One technique utilized is incorporating data collection into imagination and play. For example, in one game, children and researchers pretend to be superheroes who are frozen by villains during the 5 min sit still time and then must chew on the cotton swab to break free from the ice. Additionally, for children who struggle to focus on or comply with the study components, researchers engage them in an activity that requires them to be quietly seated (e.g., reading books, playing board games, or doing crafts at a table), allowing for a smoother transition into the sit still interval before each sample.

### 5.3. Hypervigilance

Even after the extensive training and setup process, young children and their parents may still accidentally break protocol. One key observation we have made over the years is that young children move very quickly. For example, one moment they may be sitting next to a researcher and reading, but the next moment, they “bolt” off, which increases the likelihood of a protocol violation. To mitigate potential “slip-ups”, researchers must employ hypervigilant awareness when collecting data in the home. Maintaining close proximity (within an arm’s length) to the study participant allows researchers to quickly intervene before a child can pull down a tarp covering a window or accidentally turn on an unexpected light source. Additionally, the comfort of being in the natural home environment can lead to momentary lapses for parents and participants, so close monitoring of behavior around light sources and frequent reminders about study procedures are necessary throughout the study.

### 5.4. Structure and Routine

Providing a predictable routine is beneficial to children amid the lifestyle changes that take place during the DLMO assessment. To allow for normal family eating routines throughout the assessment, parents are given a meal schedule that includes all windows of time between samples when the child may eat. Researchers also frequently encourage the child to drink water to maintain adequate saliva. Additionally, using a visual schedule with illustrated activities is a practical way to explain to the child how the day will be organized. Children choose their activities between samples, which provides a sense of autonomy over an otherwise demanding schedule. Researchers limit the choices available and avoid asking yes or no questions (e.g., “It’s time for sit still time. Do you want to color or build with blocks while we sit together?” and not “Do you want to color during our sitting time together?”, which increases the likelihood of a child saying “no”). Furthermore, designating a physical space for sample collection can strengthen the routine and help children transition into focusing on their tasks. For example, one child had a special pillow corner for sitting still labeled the “relaxation station”. Ultimately, each child has different needs, and observing and following their lead gives insight into the strategies that will produce an enjoyable and successful assessment.

Over the past 15 years, we found that these methods resulted in successful circadian assessments in > 90% of children between the ages of 2 and 5 years. We expect that this protocol would be equally successful with children throughout the school-age years. Although the basic study protocol would likely still result in an accurate assessment of DLMO in children < 2 years of age, the techniques to ensure their compliance might need to be adjusted.

## 6. Summary

In summary, conducting circadian research with young children presents several logistical and individualized challenges. The protocol and techniques described here offer ways to reduce the burden on families while maximizing child compliance, comfort, and enjoyment while participating in science. Collecting data in the cave provides an opportunity for researchers to produce ecologically valid circadian data during early childhood while maintaining a high standard of scientific rigor and well-controlled conditions.

## Data Availability

Data sharing is not applicable to this article.

## References

[1] Cajochen C., Kräuchi K., Wirz-Justice A. (2003). Role of melatonin in the regulation of human circadian rhythms and sleep. J. Neuroendocr..

[2] Lewy A.J., Wehr T.A., Goodwin F.K., Newsome D.A., Markey S.P. (1980). Light suppresses melatonin secretion in humans. Science.

[3] Duffy J.F., Wright K.P. (2005). Entrainment of the human circadian system by light. J. Biol. Rhythms.

[4] Benloucif S., Guico M.J., Reid K.J., Wolfe L.F., L’Hermite-Balériaux M., Zee P. (2005). C Stability of melatonin and temperature as circadian phase markers and their relation to sleep times in humans. J. Biol. Rhythms.

[5] Lewy A.J., Cutler N.L., Sack R.L. (1999). The endogenous melatonin profile as a marker for circadian phase position. J. Biol. Rhythms.

[6] LeBourgeois M.K., Carskadon M.A., Akacem L.D., Simpkin C.T., Wright K.P., Achermann P., Jenni O.G. (2013). Circadian phase and its relationship to nighttime sleep in toddlers. J. Biol. Rhythms.

[7] Carskadon M.A., Acebo C., Richardson G.S., Tate B.A., Seifer R. (1997). An approach to studying circadian rhythms of adolescent humans. J. Biol. Rhythms.

[8] Benloucif S., Burgess H.J., Klerman E.B., Lewy A.J., Middleton B., Murphy P.J., Parry B.L., Revell V.L. (2008). Measuring melatonin in humans. J. Clin. Sleep Med..

[9] Burgess H.J., Eastman C.I. (2005). The dim light melatonin onset following fixed and free sleep schedules. J. Sleep Res..

[10] Burgess H.J., Savic N., Sletten T., Roach G., Gilbert S.S., Dawson D. (2003). The relationship between the dim light melatonin onset and sleep on a regular schedule in young healthy adults. Behav. Sleep Med..

[11] Akacem L.D., Simpkin C.T., Carskadon M.A., Jenni O.G., Achermann P., LeBourgeois M.K. (2015). The timing of the circadian clock and sleep differ between napping and non-napping toddlers. PLoS ONE.

[12] Hartstein L.E., Behn C.D., Akacem L.D., Stack N., Wright K.P.W., LeBourgeois M.K. (2022). High sensitivity of melatonin suppression response to evening light in preschool-aged children. J. Pineal. Res..

[13] Akacem L.D., Wright K.P., LeBourgeois M.K. (2018). Sensitivity of the circadian system to evening bright light in preschool-age children. Physiol. Rep..

[14] Hartstein L.E., Behn C.D., Wright K.P., Akacem L.D., Stowe S.R., LeBourgeois M.K. (2022). Evening Light Intensity and Phase Delay of the Circadian Clock in Early Childhood. J. Biol. Rhythms.

[15] Deacon S., Arendt J. (1994). Posture influences melatonin concentrations in plasma and saliva in humans. Neurosci. Lett..

[16] Crowley S., Cain S., Burns A.C., Acebo C., Carskadon M.A. (2015). Increased sensitivity of the circadian system to light in early/mid-puberty. J. Clin. Endocr. Metab..

[17] Lewy A. (2007). Melatonin and human chronobiology. Cold Spring Harbor Symposia on Quantitative Biology.

